# An open chat between Prof Asifa Akhtar and Klaudia Jaczynska

**DOI:** 10.1002/2211-5463.13971

**Published:** 2025-01-24

**Authors:** Asifa Akhtar, Klaudia Jaczynska, Ioannis Tsagakis

**Affiliations:** ^1^ Max Planck Institute of Immunobiology and Epigenetics Freiburg Germany; ^2^ Department of Biophysics University of Texas Southwestern Medical Center Dallas TX USA; ^3^ Department of Biochemistry University of Texas Southwestern Medical Center Dallas TX USA; ^4^ Department of Pharmacology University of Texas Southwestern Medical Center Dallas TX USA; ^5^ FEBS Open Bio Editorial Office Cambridge UK

**Keywords:** academic barriers, academic initiatives, diversity, ECR support, gender representation, women in science

## Abstract

To mark the International Day of Women and Girls in Science 2025, we invited Prof Asifa Akhtar, Vice President of the Max Planck Society's Biology and Medicine section, Director at the Max Planck Institute of Immunobiology and Epigenetics in Freiburg, Honorary Professor at the Albert Ludwigs University and recipient of the 2025 FEBS | EMBO Women in Science Award to meet and chat with Klaudia Jaczynska, final year PhD student at Jose Rizo's laboratory in UT Southwestern Medical Center, Dallas, and 2024 *FEBS Open Bio* Article Prize winner. We invited them to talk about challenges limiting equal representation in science, technology, engineering and mathematics, initiatives to foster supportive environments as a research institute and the importance of highlighting diverse examples of success.

## Graphical abstract

Prof Asifa Akhtar, Director at the Max Planck Institute of Immunobiology and Epigenetics, recipient of the 2025 FEBS | EMBO Women in Science Award, and Klaudia Jaczynska, final year PhD student and 2024 *FEBS Open Bio* Article Prize winner, talk about challenges limiting equal representation in STEM, initiatives to foster supportive environments as a research institute and the importance of highlighting diverse examples of success.

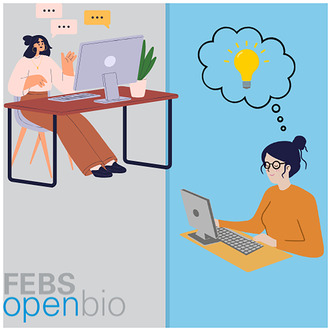

**Abbreviations**
AI: artificial intelligence
AWS: Alliance of Women Scientists
ECR: early career researcher
STEM: science, technology, engineering and mathematics

## On the challenges around equal gender representation in STEM disciplines…

**Klaudia**: From my perspective as a younger researcher, I look back and wonder, are we raising children the same way? Do boys and girls have the same opportunities, the same support system, and so on?

There is this phenomenon called ‘Mathematics Anxiety’, which tends to be observed much more in girls than in boys. Although girls perform as well as boys, at some point, the gap starts, and in part, this has to do with their attitude towards math. Of course, this can then affect which subjects women or men choose to study at university and it can also affect their confidence. For example, even though there is a high percentage of graduates in science, technology, engineering and mathematics (STEM) within women, retaining women at higher positions is much harder, so I would think that early education intervention might be needed.

I think that role models that show us what is achievable are also important, and I would like to congratulate you, Asifa, on your 2025 FEBS | EMBO Women in Science Award.

**Asifa**: The societal and cultural expectations for women and men are still quite different. I believe that a lot of progress has been made overall to showcase that men and women are equally capable, especially since at the beginning the gender distribution is always 50–50. But the bottleneck comes at every career stage as we face different challenges. Applying for a postdoc after a PhD is in principle a relatively simple decision to take, but nonetheless this is also a time where many people will have young families or think of starting a family and the practicalities can sometimes result in one partner having to sacrifice their career progression. It is often the female who will take a step back just because there are infrastructures missing like childcare etc.

There are many countries where paternity leave exists; nonetheless, even in those countries, the higher you go up the career ladder, the more difficult it is for women to get into leadership positions. This leaky pipeline is just not simply solved by infrastructure because to solve it we must get rid of our inherent biases. By setting good examples, we can enable people to see that both roles are possible and should be nurtured. But there are compromises that need to be made from both men and women to facilitate that.

Having said that I think there has definitely been some progress, especially in developed countries, and the fact we are having this conversation for me already showcases this.

**Klaudia**: Yeah, I totally agree. I think it's also important to keep having this conversation because new issues are emerging, right? For example, now with generative artificial intelligence (AI) as the existing stereotypes influence the use of this technology, the same stereotypes will be embedded in AI, which can result in perpetuating these stereotypes, so it's important to keep having this conversation and consider new variables as the world changes.

**Asifa**: We need environments which are supportive of both personal and professional growth so we can create a healthier environment. Organisations where there is gender parity, or just more diversity, generally speaking, are usually more creative because the unique aspects and experiences that come from diverse contexts can promote innovation. They are also much more fun.

## On fostering a supportive environment…

**Klaudia**: At UT Southwestern, we have a group called Alliance of Women Scientists (AWS) and our goal is to support each other and promote women leaders, not only from academia but also from industry. We invite speakers from different institutions as a way for us to find mentors, role models, people that we can look up to, and realise that even if you sometimes feel inadequate, there are other people that felt that way at a certain point but have made it.

When you were talking about parental leave, it reminded me of an example at one of the AWS meetings where one of the speakers shared that when they were preparing for their maternity leave, their PI supported them with a research assistant who kept their project alive while she was on leave. This really helped her stay active in the field without just ‘freezing’ her career during the maternal leave. That made me think that there are ways to mitigate disparity and move forward, especially for me right now as I transition from being a graduate student to a postdoc, and I'm curious about the next steps.

**Asifa**: I believe it is about nurturing an environment where people can openly talk about issues in a friendly way with each other, and in such an environment that is more proactive and creative, people will not feel as isolated. For example, if you hire internationally, you have the responsibility of creating an environment where international employees feel welcome. It's not just about putting how many international scientists you have recruited into some statistics, if at end of the day they cannot find housing and cannot cope with their everyday logistics. Specifically in academia, there are organisations, like the Max Planck Institute, that have international offices to help with visa applications and paperwork to make scientists' lives a little bit easier.

I think as organisations, we should not see the recruitment of international scientists as a challenge but rather as an opportunity because science provides the type of jobs where there are really no boundaries. Our institutes are already living examples of ‘No Barriers’ because an academic environment is totally international, and scientists are expected to travel to gain different kinds of experiences. This generates an environment in institutes that is normally very open and tolerant and that's what we should nurture.

**Klaudia**: Speaking about the isolation and institutional support, what I really like at UT Southwestern is that they really care about mental health and well‐being. We have student wellness and counselling services that are available for all the medical and graduate students and postdocs, and I know that's not the case for all the institutions, even in the US.

**Asifa**: This is where I have to say we are quite proud as an institute and as an organisation because we have mental health first‐aiders and we have courses that people can attend… We also provide people the opportunity to contact an external company if they need a sympathetic ear that's a bit more removed from the working environment. At our institutes, we also have Ombudsmen, gender equality officers, tailored courses etc.

Addressing mental stress is also part of our responsibilities as leaders. If you were in my laboratory and about to start looking for a postdoc position, I would very much want you to feel empowered to talk to me should there be anything that worried you, although I know that this can be intimidating in some cases. That's why I think that the support needs to work at different levels. If you have a good, respectful relationship with people you work with, this goes a long way to providing support, but this might not always be the case.

## On pursuing science as a career…

**Asifa**: During my postdoc time, the initial project for which I got all the funding didn't work and at that time; we were starting a new project on X chromosome regulation or epigenetic regulation in the laboratory. I just read up on it and I thought ‘Oh this is exciting’. That's when I asked my supervisor if I could go on that direction. Luckily, he was nice and forward‐looking enough to say ‘Ok, let's try it for two months’. Twenty‐five years later, I'm still working in that area. I'm so glad I had the opportunity to be in an environment where my supervisor let me change directions …Of course, you have to take your chances but it's not just luck, you have to work to enable your luck. You have to work hard and seize the opportunity when it arrives.

I think you need to be able to learn how to fail and then how to overcome it. It makes you stronger. That's why actually I'm happy that I didn't have a rosy beginning because when the opportunity came, I was ready for it.

**Klaudia**: What you're saying is really reassuring. This step by step, carrying on and building resilience. After our conversation, I already feel empowered, so, thank you for that.

**Asifa**: Happy to hear that. The most important thing is to realise that nobody's perfect. Perfection is not needed to go forward, to have a successful career. You need to be good at different things to be able to move forward. As you progress in your career, you should be able to identify where your strengths are. That's why sometimes going out of your comfort zone is the right thing to do because it will make you either understand you hate this or you will realise ‘Oh my God, I didn't realise I was good at that’.

**Klaudia**: Yeah, I love that—identifying the strengths but also the areas for improvement. For example, for me, I realised that I need to work on presenting data and communication. Even just being in front of people and giving a presentation. So, one of the ways I tried to mitigate that is by volunteering at the Museum of Modern Art, where I give tours and talk with people about art. I feel like that really accelerated my presentation skills and being comfortable in public settings.

**Asifa**: What I absolutely love, honestly speaking, is to see the changes between when the students start and when they finish in my laboratory. If you have good science, the rest will follow.

I also think we need many more examples of what a ‘successful career’ looks like because there is no perfect way and many people have gone through many diverse ways of having a career. So, we should also have EMBO PhD awards and EMBO Postdoc awards. The awards are a nice recognition but the message that they send, ‘you can also make it’, is more important than actual award itself.

## Conflict of interest

The authors declare no conflict of interest.

## Author contributions

All authors read and edited the manuscript.

